# Association of dietary inflammatory index with chronic kidney disease and kidney stones in Iranian adults: A cross-sectional study within the Ravansar non-communicable diseases cohort

**DOI:** 10.3389/fnut.2022.955562

**Published:** 2022-10-12

**Authors:** Jalal Moludi, Hawal Lateef Fateh, Yahya Pasdar, Mehdi Moradinazar, Leila Sheikhi, Amir Saber, Negin Kamari, Mitra Bonyani, Farid Najafi, Priyankar Dey

**Affiliations:** ^1^School of Nutritional Sciences and Food Technology, Kermanshah University of Medical Sciences, Kermanshah, Iran; ^2^Department of Nursing, Sulaimani Polytechnic University, Sulaimani, Iraq; ^3^Behavioral Disease Research Center, Kermanshah University of Medical Sciences, Kermanshah, Iran; ^4^Department of Nutrition, School of Allied Medical Sciences, Ahvaz Jundishapur University of Medical Sciences, Ahvaz, Iran; ^5^Medical Education Development Center, Kermanshah University of Medical Sciences, Kermanshah, Iran; ^6^Research Center for Environmental Determinants of Health, Kermanshah University of Medical Sciences, Kermanshah, Iran; ^7^Department of Biotechnology, Thapar Institute of Engineering and Technology, Patiala, India

**Keywords:** dietary inflammatory index, chronic kidney disease, glomerular filtration rate, kidney stones, cohort study

## Abstract

Chronic inflammation plays a central role in the pathogenesis of chronic kidney disease (CKD). The association of dietary inflammatory index (DII) with CKD remains underexplored. Thus, the present study aimed to determine the association between the DII, risk of CKD, and kidney stone formation using the data from the Ravansar non-communicable diseases (RaNCD) cohort study conducted in Kermanshah, Iran. The cross-sectional study was conducted using the recruitment phase data of the RaNCD cohort study comprising 9,824 individuals with an age range of 35–65 years. Food frequency questionnaires (FFQ) were used to evaluate the association between diet and DII scores. Renal function was assessed using estimated glomerular filtration rate (eGFR), blood urea nitrogen (BUN), and serum creatinine (Cr) level. CKD was defined based on eGFR. The prevalence of kidney stones was evaluated by participants’ self-report. A total of 1,791 participants (18.24%) had kidney stones, while a majority were in the first quartile (27.69%). Out of 9,824 subjects, 1,747 subjects (eGFR: 18.50 ml/min per 1.73 m2; 95% CI: 17.72–19.30) had CKD. A significant trend for eGFR across all quartiles (Qs) of DII was observed. The odds ratio of CKD in the fourth quartile (pro-inflammatory diet) was 4.38-times higher than in the first quartile (anti-inflammatory diet) of DII (95% CI = 3.58–5.36). Women were found to be more likely to have less eGFR than men in the DII Qs. Collectively, the findings indicated that consumption of a pro-inflammatory diet was associated with a high occurrence of CKD. As a matter of interest, the results also revealed that a pro-inflammatory diet had no significant correlation with kidney stone development.

## Introduction

Chronic kidney disease (CKD) is pre-epidemic on a global scale (1). Pooled estimates indicate that the prevalence of CKD ranges from 8 to 13% globally (2). Typically, since CKD affects the structure of the kidney, it progressively leads to renal dysfunction (2). The estimated glomerular filtration rate (eGFR) is direct evidence and a general measure of kidney function which lowers with CKD (3). CKD is divided into five stages by kidney disease outcomes quality initiative (KDOQI) (4) using eGFR per ml/min per 1⋅73 m^2^ as follows: stage 1 (normal or increased GFR ≥ 90), stage 2 (mildly decreased GFR 60–89), stage 3 (moderately decreased GFR 30–59), stage 4 (severely decreased GFR 15–29), and stage 5 (kidney failure, GFR < 15) (3–5).

The pathophysiological process of CKD is defined by an underlying chronic pro-inflammatory state (6, 7). Since chronic inflammation could be reflective of dietary quantity and quality, the dietary intakes in CKD patients and the regulation of chronic inflammation have been demonstrated by previous studies (8). A healthy diet rich in fibers, omega 3 fatty acids, vitamins, nuts, and fish, and containing low amounts of sodium and saturated fatty acids has been associated with improved renal function and lower hazard of albuminuria and CKD (9–11). On the contrary, the intake of simple carbohydrates, saturated fats, and trans-fats, which are in general thought to have pro-inflammatory effects, are often associated with higher rates of age-adjusted mortality in individuals with CKD (8).

The Dietary Inflammatory Index (DII) is a relatively new index that is built upon the association between diet and inflammation, appraises the overall inflammatory potentials of the dietary components, and is standardized for assessing the average dietary intake globally (12). The DII has been successfully used for several conditions including chronic systemic inflammation, obesity, diabetes, cancer, cardiovascular diseases (CVD), and fatty liver disease (12, 13).

Despite the success of DII in associating diet and inflammation with multiple diseases, research activities in related dietary habits and kidney health remain critically undertaken. Thus, understanding the role of inflammation in CKD in relation to diet can foster the development of effective prophylactic and therapeutic strategies against the underlying inflammation in CKD patients. While the inflammatory pathophysiology may not be similar in patients with chronic renal failure, persistent low-grade inflammation has been hypothesized as a risk factor for CKD (14). Dietary intake may contribute to the development of an inflammatory condition and trigger the risk of CKD (5, 9, 10). Chronic inflammation also plays an important role in the pathogenesis of kidney stone formation (15, 16). Several inflammatory biomarkers [e.g., c-reactive protein (CRP), P-selectin] usually increase in the urine of individuals with kidney stones. Furthermore, it is well-established that kidney stone formation is attributed to unhealthy dietary patterns (17) and an anti-inflammatory diet may be associated with improved kidney function (18). However, no studies have investigated the relationship between DII and kidney stone formation.

Since the onset and progression of CKD are associated with a chronic pro-inflammatory state, we hypothesized that higher DII scores, indicating a more pro-inflammatory food intake, were associated with an incidence of increased CKD and poorer clinical outcomes. Therefore, in the current study, we aimed to determine the association between DII and renal function and kidney stone formation in Ravansar non-communicable diseases (RaNCD) cohort study, Kermanshah, Iran.

## Materials and methods

### Study design

This cross-sectional study was conducted using data from the recruitment phase of a prospective study called the RaNCD cohort study. The RaNCD study is a dimension of the prospective epidemiological study in Iran (PERSIAN), which was conducted on different ethnic groups of the Iranian population in collaboration with the Ministry of Health and Medical Education, Iran. Ravansar, with a population of about 50,000, is one of the Kurdish provinces in the northwestern region of Kermanshah, Iran. The preliminary phase of the RaNCD began in November 2014 and ended in February 2017, during which 10,047 people participated in the study after informed consent. Further details and protocol of the RaNCD cohort study have been proactively published elsewhere (19, 20).

Patients with a background of cancer, thyroid disease, fatty liver, stroke, and end-stage renal disease (ESRD) were excluded due to the possibility of altered dietary patterns. In addition, subjects taking medications such as herbs, corticosteroids, and multivitamin supplements were not included in the study, as these supplements could affect nutritional outcomes for chronic renal failure. Patients with questionable total daily energy intake (less than 500 kcal/day and more than 4,200 kcal/d) were excluded from the study.

### Data collection and measurement

Information about participants in the RaNCD cohort study was gathered by a trained master at the cohort center (Ravansar cohort center).

### Assessment of renal function

CKD was characterized by renal abnormalities or a GFR < 60 ml/min/1.73 m^2^ (1.0 mL/s/1.73 m^2^) for more than 90-d. Kidney irregularities were diagnosed by pathological problems or markers of dysfunction, including abnormalities in blood or urine tests (21). Diet modification equation in kidney disease 4 (MDRD4) was used to measure eGFR using serum creatinine (Cr), age, gender, and ethnicity as follows (22):

eGFR (mL/min per 1.73 m^2^) = 175 × serum creatinine (μmol/L)–1.154 × age (years)–0.203 × 0.742 (if female) × 1.213 (if black). Abnormal stages of CKD were classified as follows: stage 3 (30 eGFR ≤ 59 mL/min/1.73 m^2^); stage 4 (15 eGFR 29 ml/min/1.73 m^2^); stage 5 (eGFR < 15 mL/min/1.73 m^2^) (23, 24).

#### Kidney stone

All the cases with kidney stones in the RaNCD cohort study by self-report were confirmed by clinical records.

#### Dietary assessment

The standardized 118-item 1-y FFQ used in a national cohort study—PERSIAN—was used to assess the dietary patterns (19). Updated nutritional databases were used to assess the amount of nutrient intake (19). The FFQ was used to determine the DII and was presented as part of the previous study (12). The DII was formulated by studying articles that were published between 1950 and 2010 and which were centered around the link between a series of food parameters and 6 inflammatory markers *viz.* IL (interleukin)-1β, IL-4, IL-6, IL-10, CRP, and tumor necrosis factor-α (TNF-α). Similarly, 45 nutritional parameters were recognized including macronutrients, micronutrients, flavonoids, and other foods, which can influence the outcomes of inflammation. The inflammatory potential of each parameter was evaluated based on their impact on the expansion, reduction, or elimination of various inflammatory markers. Foods having pro-inflammatory potentials were rated + 1, anti-inflammatory foods were rated −1, and foods with no effects on inflammation were given a score of zero. The DII values can range from −8.87 (highest anti-inflammatory score) to + 7.98 (highest pro-inflammatory score). Based on the mean admission and global SD, z-score and percentage were determined for each parameter. The inflammatory score for each of the nutritional parameters was determined and the total DII score was obtained from the set of inflammatory scores. A more negative DII score indicates the most potent pro-inflammatory diet, and a more positive DII score indicates the most impressive pro-inflammatory diet (7–12).

The nature of the diet was examined using the 2015 health eating index (HEI), which evaluates 13 food groups, including 9 adequate components and 4 moderate components. Using principal component analysis (PCA), subjects were economically categorized as poorest, middle class, richest, and rich (25).

### Clinical measurements

Diagnosis of diabetes included fasting blood glucose (FBS) levels of at least 126 mg/dL or cases treated with hypoglycemic drugs. People with a systolic blood pressure of at least 140 mmHg and diastolic blood pressure of at least 90 mmHg or who were being treated with medication for high blood pressure (BPH) were considered to have BPH. In this study, dyslipidemia was also considered a problem in serum lipid profile indices, including one or more of the following: low-density lipoprotein (LDL) > 130 mg/dL, high-density lipoprotein (HDL) < 45 mg/dL, triglycerides (TG) > 150 mg/dL, total cholesterol > 200 mg/dL, or taking lipid-lowering drugs such as amlodipine, atorvastatin, clofibrate, fenofibrate, gemfibrozil, lovastatin, and simvastatin. Blood urea nitrogen (BUN) and serum creatinine (Cr) concentrations were measured using enzymatic techniques. The one-to-one questionnaire was used to assess participants’ physical activity. The questionnaire consisted of 22 questions that assessed individual activity on an hourly or minute-per-day basis. At last, information from the questionnaire was extracted and used on the basis of metabolic equivalents (METS)/hour per day.

### Statistical analysis

Data were presented using mean ± S.D for quantitative variables and frequency and percentage for qualitative variables. Raw ORs with 95% CIs were used to analyze the relationship between DII and CKD. The relationship between the factors was assessed using univariate and multivariate logistic regression models. Variables with *P* < 0.3 in the univariate analysis were included in the multivariate model. Then, the variables with *P* > 0.05 were removed using the forward or reverse method. The fractional polynomial method was performed to quantitatively associate the effect of DII with the odds ratio of CKD. To estimate the effect of DII on CKD and kidney stones, we entered confounding and then adjusted variables for diabetes and hypertension, age, gender, smoking status, body mass index (BMI), education level, and physical activity. The effect of the DII was then evaluated. The fractional polynomial is a regular polynomial alternative method that provides flexible parameterization for continuous variables. All analyzes were performed using Stata software version 14.1 (Stata Corp., College Station, TX, United States) with a 95% confidence interval.

#### Ethical approval

The convention of this study was supported by the ethics committee of the Kermanshah University of Medical Science (IR. KUMS.REC.1394. 318).

## Results

In the present study, of the 10,047 individuals enrolled in the RaNCD cohort, the status of CKD and other related markers was recorded for 9,824 (97%) participants. Out of these, 5,214 (53.07%) were female participants. The mean ± SD age of women and men was 48.2 ± 8.3 and 47.7 ± 8.1 years, respectively. Of the participants, 2,683 (27.52%) had a normal BMI and 26.1% had low-physical activity (36 > −MET/h per day) (Table 1).

**TABLE 1:** Characteristics of the participants according to the quartiles of dietary inflammatory index.

Variable		Total	Q1 (anti-inflammatory)	Q2	Q3	Q4 (pro- inflammatory)	*P*-value*
Total (%)		9,824 (100)	2,456 (25.0)	2,456 (25.0)	2,456 (25.0)	2,456 (25.0)	
Mean (min, max)		–0.84 (–5.00, 4.64)	–2.82 (–5.00, –2.02)	–1.51 (–2.08, –1.02)	–0.45 (–1.02, 0.25)	1.40 (0.25, 4.64)	
Gender	Male	4,610 (46.93)	1,518 (32.93)	1,389 (30.13)	1,049 (22.75)	654 (14.19)	**<.001**
	Female	5,214 (53.07)	938 (17.99)	1,067 (20.46)	1,407 (26.99)	1,802 (34.56)	
Age group	35–45	4,298 (43.75)	1,172 (27.27)	1,198 (27.87)	1,074 (24.99)	854 (19.87)	**<.001**
	46–55	3,284 (33.43)	850 (25.88)	779 (23.72)	479 (21.36)	821 (25.00)	
	56–65	2,242 (22.82)	434 (25.00)	479 (21.36)	548 (24.44)	781 (34.83)	
Education level	Illiterate	2,435 (24.79)	389 (15.98)	452 (18.56)	602 (24.72)	992 (40.74)	**<.001**
	1–5 years	3,762 (38.29)	844 (22.43)	937 (24.91)	989 (26.29)	992 (26.37)	
	6–9 years	1,629 (16.58)	502 (30.82)	468 (28.73)	4,069 (24.92)	253 (15.53)	
	10.12 years	1,224 (12.46)	425 (34.72)	363 (29.66)	293 (23.94)	143 (11.68)	
	>13ψεαρσ	774 (7.88)	296 (38.24)	236 (30.49)	166 (21.45)	76 (9.82)	
Place of residence	City	5,806 (59.10)	1,912 (32.93)	1,610 (27.73)	1,344 (23.15)	940 (16.19)	**<.001**
	Village	4,018 (40.90)	544 (13.54)	846 (21.06)	1,112 (27.68)	15,169 (37.73)	
Physical Activity (MET-hours per day)	24–36.5	2,724 (24.74)	694 (25.48)	699 (25.66)	6,849 (25.11)	647 (23.75)	**<.001**
	36.6–44.9	5,073 (51.66)	1,188 (23.42)	1,209 (23.83)	1,285 (25.33)	1,391 (27.42)	
	≥45	2,023 (20.60)	573 (28.32)	547 (27.04)	485 (23.97)	418 (20.66)	
Smoking status	No	7,866 (80.27)	1,897 (24.12)	1,910 (24.25)	2,002 (25.45)	2,057 (26.15)	**<.001**
	Current	1,130 (11.53)	311 (27.52)	324 (28.67)	273 (24.16)	222 (19.65)	
	Former	804 (8.20)	239 (29.73)	216 (26.87)	177 (22.01)	172 (21.39)	
BMI (kg/m^2^)	<18.9	164 (1.68)	21 (12.80)	34 (20.73)	52 (31.71)	57 (34.76)	**<.001**
	19–24.9	2,683 (27.52)	594 (22.14)	638 (23.78)	676 (25.20)	775 (28.89)	
	25–29.9	4,241 (43.51)	1,078 (25.42)	1,115 (26.29)	1,052 (24.81)	996 (23.49)	
	30–34.9	2,087 (21.41)	574 (27.50)	519 (24.87)	509 (24.39)	485 (23.24)	
	≥35	573 (5.88)	167 (29.14)	135 (23.56)	143 (24.96)	128 (22.34)	
HEI	1st quintile (poorest)	2,071 (21.09)	143 (6.90)	342 (16.51)	585 (28.25)	1,001 (48.330	**<.001**
	2nd quintile	1,890 (19.24)	287 (15.19)	481 (25.45)	537 (28.41)	585 (30.95)	
	3rd quintile	2,115 (21.54)	497 (23.5)	579 (27.38)	582 (27.52)	457 (21.61)	
	4th quintile	1,845 (18.79)	634 (34.36)	5,499 (29.76)	414 (22.44)	248 (13.44)	
	5th quintile (highest)	1,900 (19.35)	895 (47.11)	505 (26.58)	337 (17.74)	163 (8.58)	
Kidney stone	No	8,025 (81.74)	1,958 (24.40)	2,030 (25.30)	2,001 (24.93)	2,036 (25.37)	**0.01**
	Yes	1,791 (18.24)	498 (27.69)	424 (23.67)	418 (23.34)	418 (23.34)	
Type 2 diabetes	No	8,964 (91.79)	2,239 (24.98)	2,243 (25.02)	2,236 (25.06)	2,246 (25.06)	**0.98**
	Yes	802 (8.21)	200 (24.94)	198 (24.69)	205 (25.56)	199 (24.81)	
Hypertension	No	8,251 (84.18)	2,111 (25.58)	2,126 (25.77)	2,043 (24.76)	1971 (23.89)	**<.001**
	Yes	1,551 (15.82)	341 (21.99)	328 (21.15)	405 (26.11)	477 (30.75)	
eGFR	Mean ± SD	76.1 ± 14.1	80.0 ± 13.4	78.3 ± 14.3	75.5 ± 13.7	70.7 ± 13.4	**<.001**
Creatinine (mg/dL)	Mean ± SD	0.99 ± 0.18	0.98 ± 0.17	0.99 ± 0.19	0.98 ± 0.17	0.99 ± 0.17	**0.9**
Urea	Mean ± SD	13.56 ± 4.2	13.91 ± 4.1	13.7 ± 4.1	13.3 ± 3.9	13.2 ± 2.3	**0.4**

*p-value < 0.05 were considered statistically significant.

The average DII score In this study was −0.84 ± 1.6, ranging from −5.00 (diet with the lowest pro-inflammatory potential) to + 4.64 (diet with the highest pro-inflammatory potential). Table 1 summarizes the general characteristics of subjects with CKD in the DII Qs. There was no significant difference in the prevalence of diabetes (*p* = 0.98) between the DII Qs.

According to the participants’ self-reported data, 18.24% of them had kidney stones, and most of them (27.69%) were in the first quarter. In addition, as the inflammation of the diet increased, a decreasing trend in the incidence of kidney stones was observed among the study participants. However, no significant differences were found in the incidence of kidney stones between the quartiles (*P* = 0.01) (Table 1).

In this study, out of 9,824 subjects 1,747 subjects [eGFR: 18.50 mL/min per 1.73 m2; 95% confidence interval (CI): 17.72–19.30] had CKD. The prevalence of kidney stones was evaluated by participants’ self-report. A total of 1,791 participants (18.24%) had kidney stones, and most of them were in the first quartile (27.69%).

The highest prevalence of kidney stones was observed in the first quartile (diet with the highest anti-inflammatory potential). No significant differences were found in creatinine and urea concentrations across the Qs or DII score (Table 1).

**FIGURE 1:**
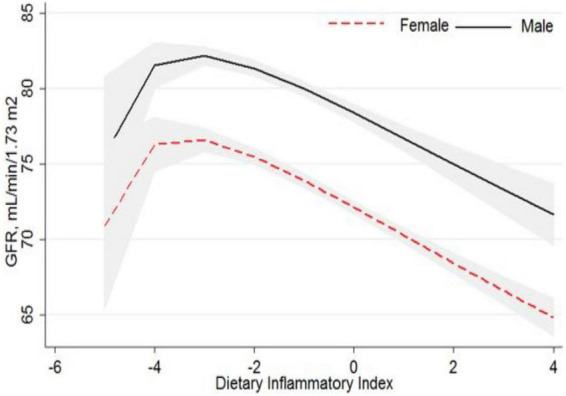
Association of glomerular filtration rate and dietary inflammatory index.

The average eGFR in men (80.2 ± 13.1) was higher than in women (72.6 ± 13.5) in the study population. Figure 1 shows the mean of eGFR over Qs of DII. A significant decreasing trend was observed for eGFR across Qs of DII in men and women. Subjects with a higher DII had lower eGFR and the trend was similar in both genders.

It is likely that a pro-inflammatory diet increases the risk of developing CKD. According to our findings, which can be seen in the crude model, the risk of CKD in quartile 4 was 4.38 times higher than that in quartile 1 (OR = 4.38, 95% CI: 3.58–5.36). Although the odds ratio decreased compared to the crude model after controlling for confounding factors, the association between DII and CKD remained significant. Moreover, after adjusting for all major confounding factors, the probability of chronic renal failure in subjects in the fourth quartile (pro-inflammatory) was 1.92 (95% CI: 1.52–2.42) times higher than Q1 (anti-inflammatory) (Table 2).

**TABLE 2:** Bivariate and the multivariate association between DII and CKD.

Variable	Mean (min-max)	Crude	Model 1*	Model 2^†^	Model3^‡^
		OR (CI 95%)	OR (CI 95%)	OR (CI 95%)	OR (CI 95%)
DII	Q1 (anti-inflammatory)	1	1	1	1
	Q2	1.39 (1.10–1.75)	1.25 (0.98–1.59)	1.13 (0.98–1.44)	1.14 (0.89–1.45)
	Q3	2.31 (1.86–2.86)	1.75 (1.40–2.19)	1.48 (1.17–1.87)	1.44 (1.14–1.82)
	Q4 (pro-inflammatory)	4.38 (3.58–5.36)	2.49 (2.01–3.08)	1.95 (1.54–2.46)	1.92 (1.52–2.42)

*Model 1: Adjusted for baseline age, gender, smoking status, BMI, place, education level, and physical activity.

^†^Model 2: In addition, adjusted for HEI.

^‡^Model 3: In addition, adjusted for kidney stone, diabetes, and high blood pressure.

**FIGURE 2:**
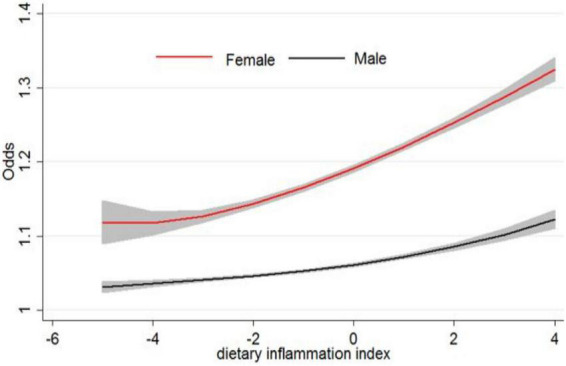
Odds of chronic kidney disease and dietary inflammatory index.

As shown in Figure 2, an increased risk of being in the higher stage of CKD was found among those in the top Qs of DII (*P* for trend = 0.03). The results also revealed that women were more likely to get CKD than men.

## Discussion

The present study was conducted in an Iranian Kurdish population to assess the relationship between DII and the likelihood of CKD progression in a large general population in Iran. We have shown that DII scores are directly related to CKD risk. The odds ratio of CKD in the fourth quarter was 4.38 times higher than in the first quarter of DII. These findings remain in accordance with prior studies (11, 15, 26). Mihai et al. indicated that in patients with CDK, consuming a diet with pro-inflammatory potential may trigger disease progression (15). Another observational study examining the likelihood of harmful effects of pro-inflammatory diets on kidney health suggested that the anti-inflammatory properties of the diet are crucial for preventing kidney failure in American adults (27). A cohort study recommended that a pro-inflammatory diet, as assessed by the DII, was associated with systemic inflammation and impaired renal function in older Swedish populations (11). The significant mediating role of serum CRP in the association between the DII and kidney function proposes that inflammation is a likely mechanism through which diet may prompt kidney dysfunction (11). Therefore, an anti-inflammatory diet appears to be a reasonable prophylactic strategy for limiting the risk of CKD. Overall, previous evidence has shown that diet quality can develop outcomes in patients with chronic renal failure (28). A pro-inflammatory diet is not only effective in reducing the burden of disease in patients with chronic kidney failure but also in preventing kidney cancer. Shivappa et al. showed that higher DII scores for pro-inflammatory diets were associated with a higher risk of kidney cancer in the American population (29). An anti-inflammatory diet high in fruits and whole grains was associated with lower urinary albumin to creatinine ratio (ACR) (30), whereas a pro-inflammatory diet such as animal-based foods was associated with higher ACR across all quartiles (31). Furthermore, a greater intake of animal fats and sodium is associated with the onset of micro albuminuria, while a greater intake of carotenoids, which have an anti-inflammatory effect, is associated with an increase in GFR (32). One of the potential mechanisms adding to the relationship between DII and the risk of renal disease is the impact of diet-related chronic inflammation in the upregulation of various pro-inflammatory mediators like TGF-β, TNF-α, IL-6, and CRP (14). In contrast, others have shown that the DII score is unrelated to serum hs-CRP and biomarkers of kidney function in elderly patients (26). Nevertheless, previous reports suggest that dietary habits are associated with CRF according to major community registries (9). The DII has a good overall ability to assess nutritional and inflammatory status to reduce morbidity and mortality in patients with chronic renal failure (33).

In the present study, we also observed significantly fewer kidney stones in subjects in Q1 of the DII compared to the later Qs. It has been suggested that the formation of kidney stones could be attributed to poor dietary habits (16, 17). An earlier study showed that adherence to an unhealthy dietary pattern that is rich in red meats and high-fat dairy products is related to kidney stone formation (17). Moreover, adherence to the Mediterranean dietary pattern (18) and DASH (dietary approaches to stop hypertension) diet (16) with a low DII, which includes a high intake of fruits, vegetables, and low-fat dairy products and a low intake of total fat, are associated with decreased kidney stone formation. Earlier studies likewise propose that individuals with kidney stones are inclined to CKD at later stages of life (34, 35).

Our study showed that there was no significant difference in the prevalence of diabetes between the Qs of the DII. In contrast to this study, Nikniaz et al. showed that the DII score was related to total metabolic syndrome (MetS) and FBS after adjusting for all covariates in Iranian adults (36). In a cross-sectional study, the upper DII quartile (Q4) was positively associated with the prevalence of MetS in men and in postmenopausal women (37). Another study showed that an increased pro-inflammatory diet is associated with poor glucose homeostasis (38).

Despite the fact that hypertension and CKD coexist frequently (39), findings of our study demonstrated that patients with HBP as the highest consumer of an anti-inflammatory diet. This finding does not corroborate any previous study. Phillips et al. proposed that expanded admission to a pro-inflammatory diet is related to higher blood pressure among an Irish population (38). Ramallal et al. likewise showed a significant association between DII and hypertension in a Spanish population (40). Our results also showed that women had a higher risk of CKD progression than men consistent with a previous study (41). It has been proposed that the distinction in sexual orientation in CKD can be largely attributed to differences in predictors of renal function due to urinary tract infection, especially in women (42).

At last, our study consists of certain limitations. First, we calculated the DII using 29 diet items, and data on 16 diet items were not available in this study. Second, due to the cross-sectional design of the present study, it was not possible to investigate the causal relationship between DII and the progression of CKD. Nevertheless, the greatest strength of the present study was that it aimed to determine the relationship between DII and CKD among the Kurdish population in Iran with large sample size. Other strengths of this study are the high quality of data collection, population-based study design, and adjustment for all known confounders such as age, sex, smoking status, BMI, location, level of education, and physical activity. Using DII instead of inflammatory markers to assess the effect of inflammation may help directly measure the impact of diet on clinical outcomes through inflammation and reduce the overall cost of the study. The calculation of the DII by an inexpensive and non-invasive method (FFQ) makes it possible to evaluate the inflammatory properties of the dietary components.

## Conclusion

In conclusion, the lowest quartiles of DII were at a reduced risk of being in the highest stage of CKD and improved renal function in a large general population. Considering the role of diet through its antioxidant properties in the occurrence of diseases such as CKD, it is recommended that the DII should be taken into account to help prevent, control, and treat CKD, with an emphasis on the use of antioxidant diets as part of prophylactic dietary strategies.

## Data availability statement

The raw data supporting the conclusions of this article will be made available by the authors, without undue reservation.

## Ethics statement

Written informed consent was obtained from the individual(s) for the publication of any potentially identifiable images or data included in this article.

## Author contributions

YP, NK, and FN designed the study. YP completed the entire study. JM and LS collected and analyzed the data. JM and HF prepared the manuscript. MM conducted the statistical analysis. All of the authors edited the manuscript.
